# A preliminary study of ALPPS procedure in a rat model

**DOI:** 10.1038/srep17567

**Published:** 2015-12-03

**Authors:** Huawen Shi, Guangchao Yang, Tongsen Zheng, Jiabei Wang, Lulu Li, Yingjian Liang, Changming Xie, Dalong Yin, Boshi Sun, Jing Sun, Huanlai Wang, Shangha Pan, Hongchi Jiang, WanYee Lau, Lianxin Liu

**Affiliations:** 1 Key Laboratory of Hepatosplenic Surgery, Ministry of Education, Department of General Surgery, the First Affiliated Hospital of Harbin Medical University, Harbin, China; 2 Department of MRI, the First Affiliated Hospital of Harbin Medical University, Harbin, China; 3 Department of General Surgery, Qiqihaer City Hospital of Traditional Chinese Medicine, Qiqihaer, China; 4 Faculty of Medicine, the Chinese University of Hong Kong, Prince of Wales Hospital, Shatin, New Territories, Hong Kong SAR, China

## Abstract

Associating Liver Partition and Portal vein ligation for Staged hepatectomy (ALPPS) has been reported to be a novel surgical technique that provides fast and effective growth of liver remnant. Despite occasional reports on animal studies, the mechanisms of rapid liver regeneration in ALPPS remains unclear. In the present study, we intend to develop a reproducible rat model to mimick ALPPS and to explore the underlying mechanisms. Rats assigned to the portal vein ligation (PVL), left lateral lobe (LLL) resection, transection and sham groups served as controls. Results indicated that the regeneration rate in the remnant liver after ALPPS was two times relative to PVL, whereas rats with transection alone showed minimal volume increase. The expression levels of Ki-67 and PCNA were about ten-fold higher after ALPPS compared with the transection and LLL resection groups, and four-fold higher compared with the PVL group. The levels of TNF-α, IL-6 and HGF in the regenerating liver remnant were about three-fold higher after ALPPS than the controls. There was a more significant activation of NF-κB p65, STAT3 and Yap after ALPPS, suggesting synergistic activation of the pathways by PVL and transection, which might play an important role in liver regeneration after ALPPS.

Hepatic resection is still one of the most effective treatments for liver tumors^1^. However, the extent of hepatic resection is limited by the minimum volume of the future liver remnant (FLR), which is required to provide sufficient postoperative liver function^2^. Extended hepatectomy can lead to clinical manifestations, like “post-hepatectomy liver failure” (PHLF) and “small-for-size syndrome” (SFSS). To reduce the risk of PHLF or SFSS in patients with a marginal FLR, portal vein ligation (PVL) or portal vein embolization (PVE) has been widely used to increase the FLR volume^3^,^4^. However, the drawbacks of these procedures lie in the need to wait for adequate FLR, which can be two to eight weeks, with disease progression in the meantime^5^,^6^.

The associating liver partition and portal vein ligation for staged hepatectomy (ALPPS) procedure is a new surgical strategy. It represents a modification of a two-stage hepatectomy in which complete parenchymal transection is associated with ligation of contralateral portal vein^7^,^8^. It produces a significant increase of the FLR in a much shorter time compared with PVL or PVE. Since the first report by Baumgart^9^, an increasing number of cases have been published, gaining wide interest from all over the world^10^. Although it is clear that ALPPS can induce accelerated liver regeneration, the underlying mechanisms remain unclear, and they need well-designed animal studies to clarify. To the best of our knowledge, there are only few studies on animal models for the ALPPS procedure^11^,^12^,^13^. Schlegel *et al.* developed a reproducible mouse model mimicking ALPPS and explored the underlying mechanisms. Their results suggested that ALPPS in rats induces an unprecedented degree of liver regeneration, comparable with humans. PVL in combination with the circulating factors seem to mediate the enhanced liver regeneration after ALPPS^11^. The other two groups developed a rat model of ALPPS, but they only set up Step I of ALPPS, and started to preliminarily investigate the underlying mechanisms^12^,^13^. The aims of the present study were to study liver regeneration in rats after ALPPS including both Step I and Step II and to explore the underlying mechanisms of rapid liver regeneration after ALPPS.

## Results

### Anatomy of the rat liver and successful establishment of the ALPPS animal model (Step I)

**Figure 1:**
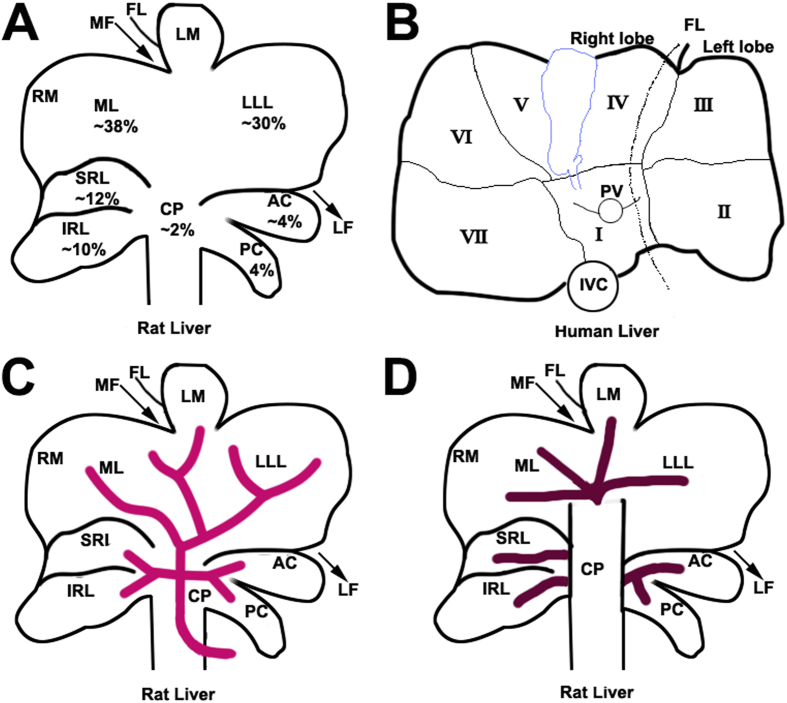
Anatomy of rat liver and human liver. (**A**) Visceral surface of the rat liver showing lobes and their mean relative weight. The caudate lobe (CL) is formed by the CP, AC and PC, the right liver lobe is formed by SRL and IRL and the medial lobe formed by LML and RML. (**B**) Visceral surface of a human liver showing division into segments according to Couinaud’s nomenclature. Most common anatomy of the portal vein (**C**) and hepatic veins (**D**) of the rat. In the rat, CL, LLL, LML, RML, (IRL + SRL) represent the human segments I and IX, segment II, segments III, IV, V and VIII; and segments VI and VII, respectively. In the rat, the left hemi-liver consists of the LLL, CL and LML while the right hemi-liver represents RL (SRL + IRL) and the RML. CP, caudate process; AC, anterior caudate lobe; PC, posterior caudate lobe; SRL, superior right lateral lobe; IRL, inferior right lateral lobe; ML, median lobe; RML, right portion of the medial lobe; LML, left portion of the medial lobe; LLL, left lateral lobe; MF, median fissure; LF, left fissure; RF, right fissure and FL, falciform ligament; PV, portal vein and IVC, inferior vena cava. (Fig. 1 was drawn by Tongsen Zheng.)

**Figure 2:**
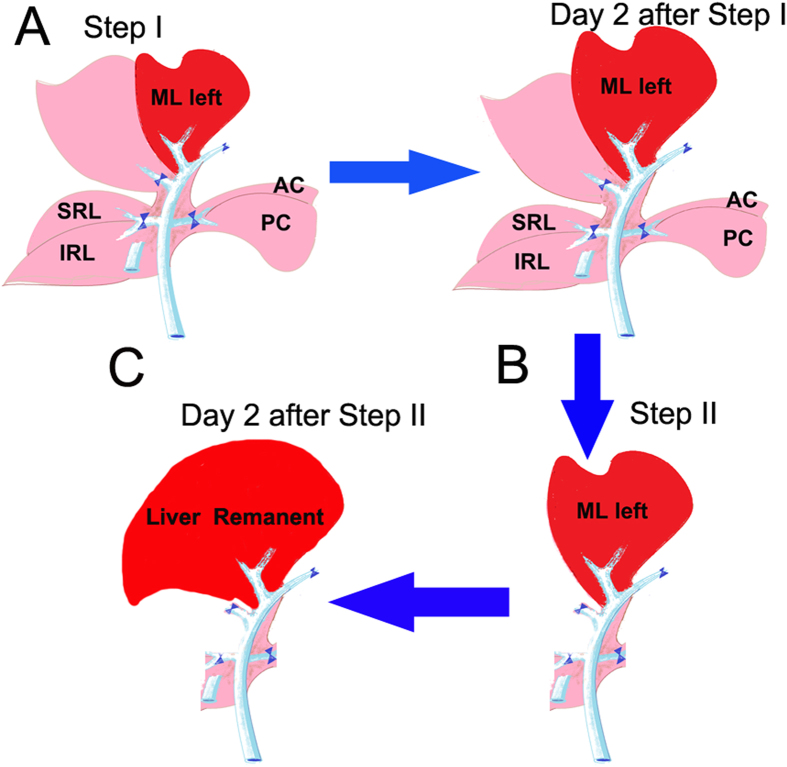
Illustrations of surgical procedure of ALPPS in the present study. (**A**) Step I of ALPPS in rats; PVL performed at the caudate lobe, right lobe, and right median lobe. Note the demarcation between normally perfused left median lobe and portal-depleted right median lobe, where transection is performed. (**B**) Two days after step I, the left median lobe shows significant increase in size, and step II including resection of all deportalized liver lobes is performed. (**C**) Again, 2 days after step II, the remnant liver shows a significantly increased regeneration. (Fig. 2 was drawn by Tongsen Zheng.)

**Figure 3:**
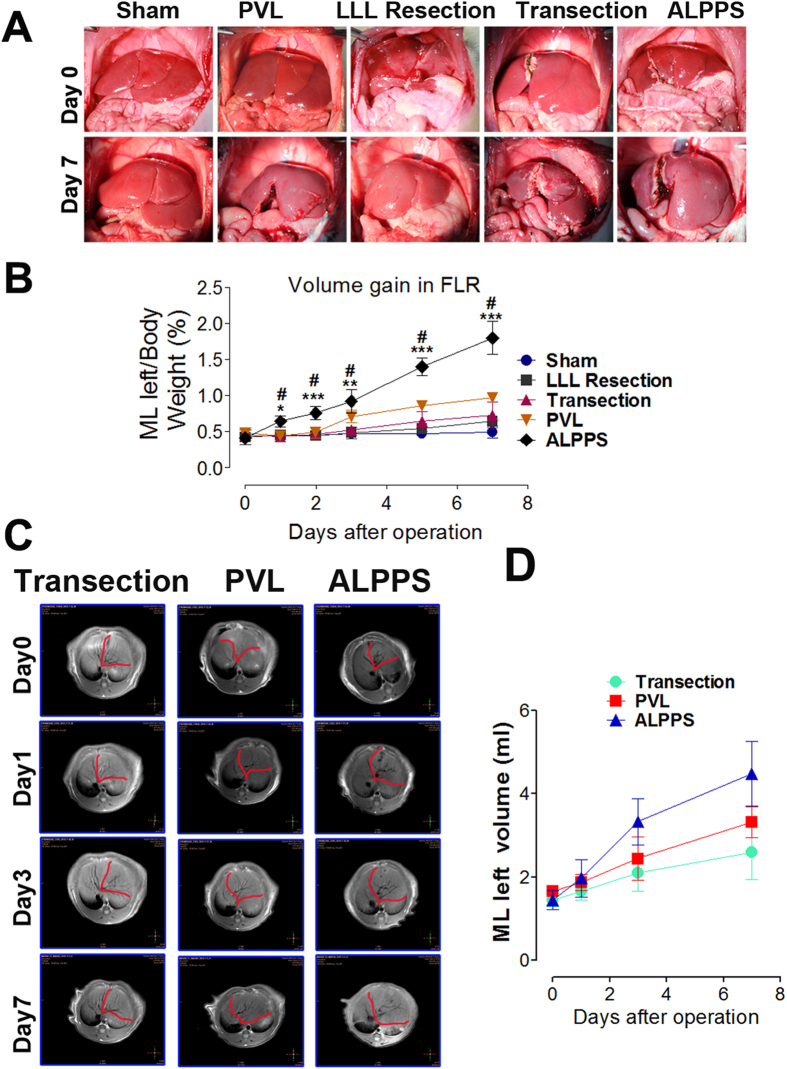
Liver volume and weight changes of the rats after surgery interventions in different groups. (**A**) Represent images of the liver on day 0 and after operations on day 7. (**B**) Changes in left side of median liver lobe weight/body weight ratios after operations in rats. Means ± SD. (n = 6 animals per group at each time point). *P < 0.05, **P < 0.01, ***P < 0.001 compared with the ALPPS group, #P < 0.001 compared with the LLL resection, transection and sham groups. (**C**) Represent MRI coronal reformatted images of the rat liver after different operations. (**D**) Time course of MRI liver absolute volumes (LML) prior to (day 0) and immediately after (day 1) surgery, and for 7 days thereafter. Values are means ± SD.

The rat liver is divided into the right lobe, median lobe, left lateral lobe, and caudal lobe (Fig. 1A). Each lobe is supplied by its individual pedicle portal(s) and drained by hepatic vein(s) (Fig. 1B,C). The median lobe is supplied by two portal branches: the right branch and the left branch. On this basis, the experimental model of ALPPS was developed and the anatomy of the rat liver was modeled after the human liver (Fig. 1D) to develop thetecnica de ALPPS. ALPPS procedure in rats as described in the Methods Section and in Fig. 2. Most rats tolerated the operative procedures well and recovered uneventfully from anesthesia, with a survival rate of 86% after surgery. The low mortality suggests that ALPPS in rats does not commonly induce liver failure, despite 90% PVL combined with liver transection. There were no postoperative ascites or bleeding complications on the raw liver surfaces (Fig. 3A).

### Liver regeneration in the left side (LML) and atrophy in the right side (RML) of the median liver lobe after ALPPS Step I

**Figure 4:**
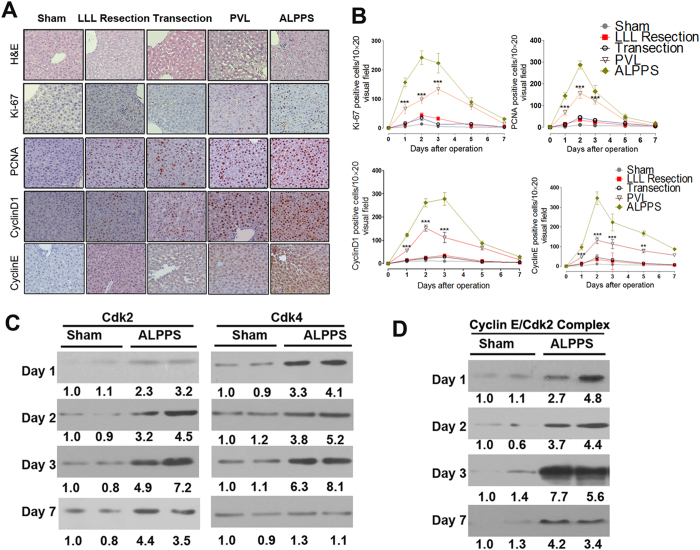
H&E staining, proliferation markers and cell cycle regulators in future liver remnants after ALPPS during the first week. (**A**) Represent images of the H&E staining, Ki-67, PCNA, cyclin D1 and cyclin E histological staining of the regenerating lobe after the procedures on day 3. (**B**) The number of positive hepatocytes per visual field (200×) is presented. Values are means ± SD. **P < 0.01, ***P < 0.001, indicated the increase of positive hepatocytes show significant differences in ALPPS versus the other three groups. (**C**) Western immunoblot analysis of Cdk2 and Cdk4 from future liver remnant on day 1, 2, 3, and 7 after either Sham or ALPPS. The densitometric analysis of Western blots were shown as percent of time-matched Sham controls. (**D**) Cyclin E/Cdk2 complex formation after ALPPS and the densitometric analysis of Western blots were shown. The immune complexes were resolved by SDS-PAGE, transferred to Immobilon membrane, and immunoblotted with anti-Cdk2.

Liver regeneration due to different surgical procedures was compared. The weight ratio of the LML to body weight before surgery was around 0.41 ± 0.04**%.** In the ALPPS group, it increased to 0.92 ± 0.11% (about 2.3 times the preoperative value) on day 3, reaching 1.81 ± 0.23% (an increase of about 3.41 times compared to preoperative values) on day 7 (Fig. 3B). For the LLL resection, transection and PVL groups, the ratios increased by 52.4%, 62.2%, 100.02% respectively on day 7. The liver regeneration was also measured by MRI assays as shown in Fig. 3C,D. Taken together, these results indicated that ALPPS induced a greater and faster liver regeneration response compared with the other groups. To further characterize the regenerative response, two nuclear antigen markers associated with cell proliferation, Ki-67 and PCNA, were evaluated. Results indicated that Ki-67 and PCNA were expressed in numerous hepatocytes in the ALPPS group (Fig. 4A,B). The PVL group exhibited less proliferative activities and the LLL resection and transection groups even much less proliferative activities. Furthermore, the atrophy of the RML was also determined. The weight ratio of the RML to the body weight decreased by 65.1% and 57.4% in the ALPPS and PVL groups, respectively, while there was no significant changes in the transection group and only a slight increase in the LLL resection group (Fig. S1). The initial and final body weights of the animals in the study were also determined. The results reflected a good consistency with regeneration of the liver weight (Fig. S2).

### Liver regeneration is associated with marked increases of the expression and function of cell cycle regulators after ALPPS

As reported, liver regeneration is characterized by a synchronous induction of normally quiescent hepatocytes to reenter the cell cycle^14^. Herein, we also investigated the effects of the different procedures on cell cycle regulators. IHC results indicated that ALPPS stimulated cyclin D1 and cyclin E expression more significantly compared with other procedures and the maximum induction occurred on day 3 and day 2, respectively (Fig. 4A,B). The cyclin D1 level returned to sham levels by 7 days, whereas the cyclin E expression remained elevated up to 7 days after ALPPS. The expression of G1 Cdks were examined by Western blot (Fig. 4C). Cdk2, the catalytic partner of cyclin E, was induced at 24h after ALPPS; the level of Cdk2 remained elevated throughout the regenerative period. Cdk4, the catalytic partner of cyclin D1, was also induced beginning 24 h after ALPPS, with a maximum induction on day 3 compared with sham group. Activation of Cdk2 kinase requires binding of cyclin E and is essential for cells to enter S phase. Because both cyclin E and Cdk2 are induced after ALPPS, we next examined whether the cyclin E/Cdk2 complex accumulated in ALPPS animal livers. Immune complexes were precipitated with antibodies against cyclin E, and the precipitates were resolved by gel electrophoresis and probed with anti-Cdk2 antibodies to measure cyclin E-associated Cdk2. As shown in Fig. 4D, formation of the cyclin E/Cdk2 complex was induced 24 h after ALPPS, with a maximum induction occurring on day 3. Next, we examined Cdk2-associated kinase activity during liver regeneration. Cell extracts were immunoprecipitated with either anti-Cdk2 antibody, and the immune complexes were assayed for kinase activity by using histone H1 as a substrate (Fig. S3). A significant increase in histone H1 kinase activity was noted 24 h, 48 h and 72 h after ALPPS compared with Sham; the Cdk2 activity returned to Sham levels by 7 days. Collectively, these results demonstrated not only temporal increases in the cyclin E/Cdk2 complex, but also concomitant increases in Cdk2 kinase activity during hepatocyte proliferation after ALPPS.

### Liver Injury and Biochemical Assay of Blood Sample

**Figure 5:**
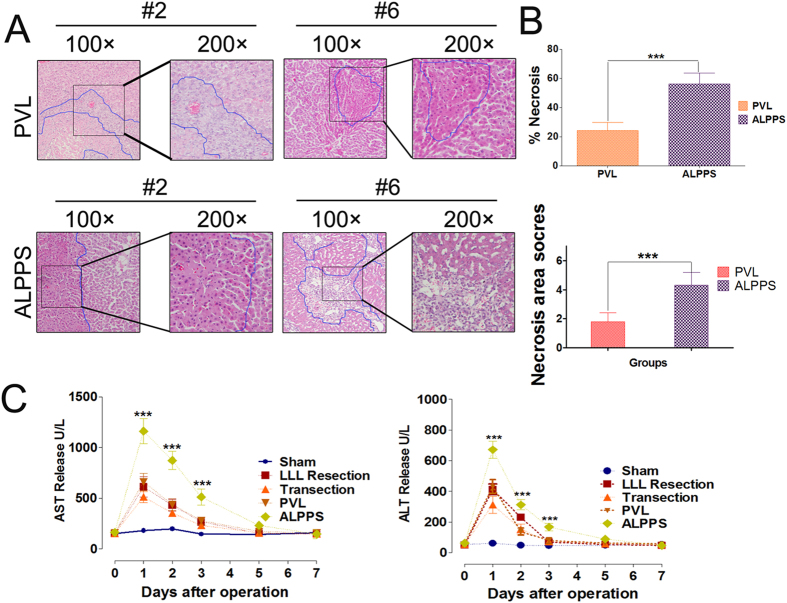
Quantification of hepatocellular injury after different surgical interventions. (**A**) Represent H&E staining of perivascular necrosis in liver lobes after PVL or ALPPS at the 24 h time point (100× and 200×). (**B**) The necrosis areas pervisual field (100×) of the RML in ALPPS was significantly different compared with that in the PVL group at 24 h (*P < 0.001). Values are means ± SD. (**C**) AST and ALT release at all time points (after ALPPS Step I surgery) were measured. There was a significant difference in AST and ALT release between the ALPPS and the other groups on day 1, day 2 and day 3 (*P < 0.001). Values are means ± SD.

To further test whether these surgical procedures caused liver injury, serum aminotransferase (AST and ALT) levels as established markers of hepatocyte injury were measured and the part of the occluded RML was examined using H&E staining. Histological studies after the PVL and ALPPS revealed areas of necrosis in the occluded part of the RML, which were significantly larger after ALPPS than after PVL on day 2 (Necrosis percentage, 56.4 ± 7.2% versus 24.3 ± 5.8%; necrosis score, 4.23 ± 0.98 versus 1.86 ± 0.64) as shown in Fig. 5A,B. The other groups did not show any area of necrosis. The LLL resection, transection and PVL groups showed comparable increase in AST and ALT levels on day1, day 2 and day 3 after Step I, which were lower than the ALPPS group (Fig. 5C). There were no significant differences among these five groups on day 5 and day 7.

### Alterations in proteins during liver regeneration after different surgical procedures

**Figure 6:**
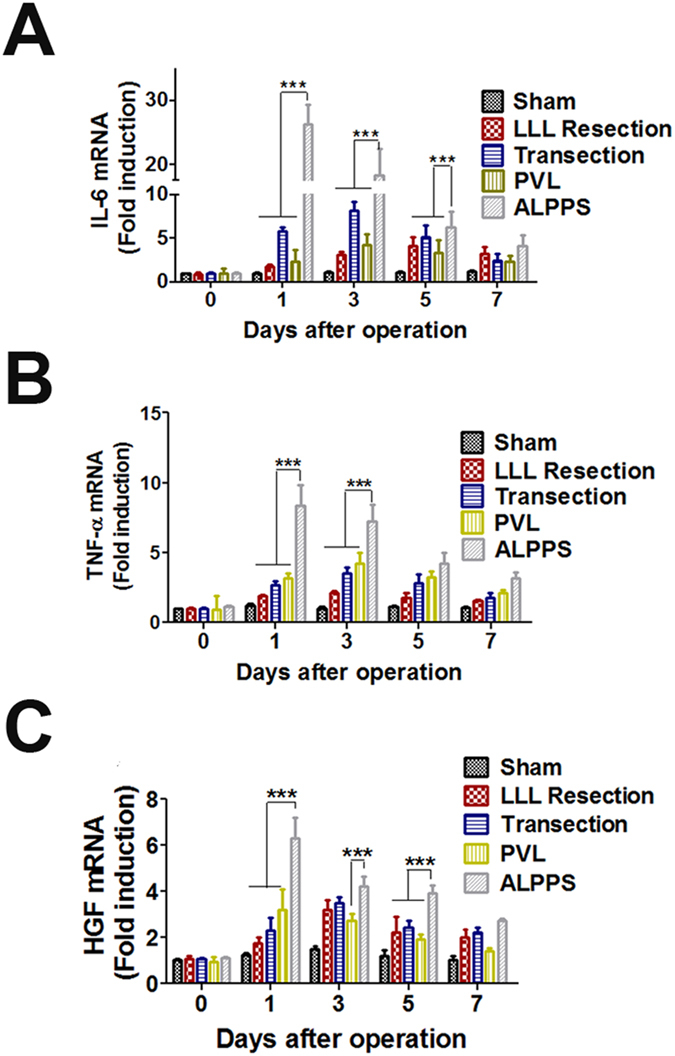
Effects of surgical interventions on the expression of inflammatory mediators and regeneration-related factors. The relative mRNA levels of IL-6 (**A**), TNF-α (**B**), and HGF (**C**) were determined by Real time-PCR and given as fold inductions relative to the sham-operated livers. (***P < 0.001 compared with the other three groups).

To investigate the mechanisms of liver regeneration after different surgical procedures, the expression changes of liver regeneration relative genes were investigated using ELISA (Table S1). Results indicated the levels of IL-6, Nuclear factor-κB p65 (NF-κB p65), signal transducers and activators of transcription 3 (STAT3), TNF-α, EGF, HGF, ERK-1/2 and YAP were significantly increased in the ALPPS group compared with the other groups, suggesting that these factors might play important roles in the fast liver regeneration after ALPPS. The release of established mediators of liver regeneration, including TNF-α, IL-6 and HGF^14^, was then confirmed by Real-time PCR (Fig. 6A–C). As expected, the mRNA levels of these cytokines were highly increased in the regenerating lobes 24 h, 3 days and 5 days after surgery in the 4 surgery groups when compared with the sham group. Importantly, there was a more significant increase of these cytokines in the ALPPS group when compared with the other three groups.

### NF-κB p65, STAT3, and YAP are most significantly induced after ALPPS procedure

**Figure 7:**
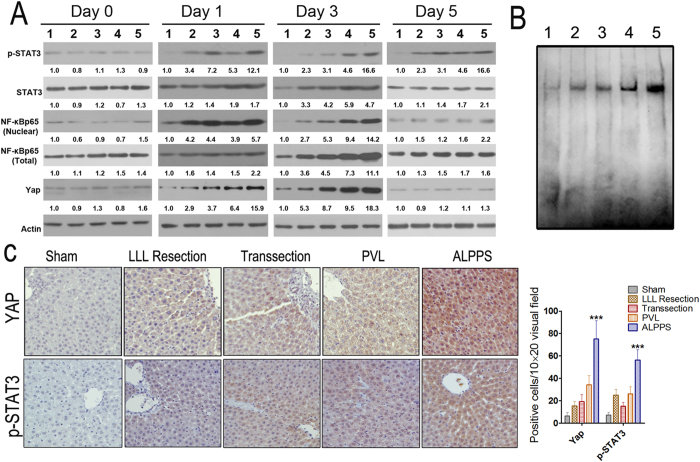
Effects of surgical interventions on the expressions of regeneration-related signaling pathways. (**A**) Protein levels of p-STAT3 (Tyr705), STAT3, Yap, nuclear and total NF-κB p65 in regenerating liver tissue at different time points post surgery were determined by western blotting. (1, sham; 2, transection; 3, LLL resection; 4, PVL; 5, ALPPS) (**B**) NF-κB DNA binding activity by EMSA in the regenerating liver tissues. Five micrograms of nuclear protein were used to analyze DNA binding activity by EMSA as described in Materials and Methods. (1, sham; 2, transection; 3, LLL resection; 4, PVL; 5, ALPPS) (**C**) Protein levels of Yap and p-STAT3 (Tyr705) in regenerating liver tissues on day 1 after different surgical interventions were determined by IHC. (***P < 0.001 compared with the other three groups).

As previously reported, studies of gene-expression cascades on the molecular mechanisms in the regenerating liver have provided insights into the signaling pathways that are rapidly activated in the liver remnant after hepatectomy or PVL^15^,^16^. In the study, changes of several specific transcription factors including NF-κB p65 and STAT3 in livers were studied. The Real-time PCR results indicated that the mRNA levels of NF-κB and STAT3 were similarly induced in the LLL resection, transection, and PVL groups on day 1 and day 2, but the induction of these factors were most significant in the ALPPS group (Fig. S4). The mRNA levels of NF-κB and STAT3 returned to sham levels by day 5. The protein expression changes of nuclear NF-κB p65 and p-STAT3 (Tyr705) were consistent with the mRNA changes on day 1 and day 2, which were most significant in the ALPPS group than the other groups (Fig. 7A). The nuclear NF-κB p65 level almost returned to sham levels by day 5, however, the level of p-STAT3 (Tyr705) remained elevated until day 5 (Fig. 7A). The results of activation of NF-κB p65 which was investigated using EMSA indicated that the activation was most obvious in the ALPPS group when compared with the other groups (Fig. 7B). Interestingly, we found that although there is an slight increase of total NF-κB p65 and STAT3 on day 1, the levels of them were only significantly induced beginning 2 days after the procedures, and returned to sham levels by day 5, which suggested there might be a delay in protein expression changes of STAT3 and NF-κB p65 compared with the mRNA levels. Recently, the Hippo-YAP signaling pathway has been implicated in liver regeneration^17^,^18^,^19^, but its role in liver regeneration after ALPPS remains unknown. The western blotting results indicated that its expression increased significantly on postoperative day 1 and day 2. Importantly, the increase was most significant in the ALPPS group. The IHC analysis of YAP and p-STAT3 (Tyr705) in the regenerating liver at 24 h following surgery also suggested the strongest staining in the ALPPS group (Fig. 7C).

### The establishment of the ALPPS procedure—Step II

**Figure 8:**
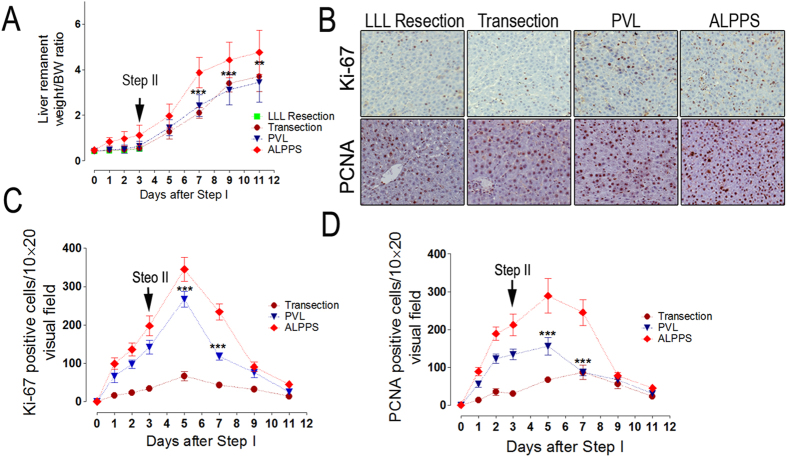
Volume gain and regeneration markers after completion of ALPPS procedure—Step I and II in rats. (**A**) Liver volume combining steps I and II at different time points. Three days after step I, we performed step II, the ALPPS rats displayed an accelerated liver regeneration as compared with rats with PVL or transection alone. Following 9 days of observation after step II, LR after ALPPS terminated growth when basically a new liver was grown (with a liver/body weight ratio of a normal liver: 4.63 ± 0.65% in our experiments). (**B**) Represent images of the Ki-67 and PCNA histological staining of the regenerating lobe after the procedures on day 2 after Step II. (**C,D**) The number of positive hepatocytes per visual field (200×) is presented. ***P < 0.001, ALPPS vs PVL and transection.

A major advantage of ALPPS is the possibility for an early completion of Step II in clinical practice, that is, removing the deportalized liver within 2 weeks after Step I. In our study, seven animals which were allocated to the ALPPS group showed a normal liver remnant to bodyweight ratio (LR/BW ratio) 2 days after Step II or 9 days after Step I. This fast rate of liver regeneration was consistent with a previous report^11^. The sizes of the remnant livers (LML) were comparable with the sizes of the original livers (Fig. 8A). As expected, all the animals allocated to the sham and the LLL resection groups died from PHLF and SFSS within 36 hours after Step II as no effort was taken to induce liver regeneration in this group of animals in Step I. Of note, Step II was feasible in all the other surgical groups and led to 90% survival after ALPPS, PVL, or transection. The expressions of Ki-67 and PCNA were also determined after Step II and the results were expected (Fig. 8B–D). In addition, the expressions of liver regeneration related proteins were investigated using ELISA on day 2 after the Step II of ALPPS, and the results were similar to the results on day 2 after Step I (Table SI).

## Discussion

The size of the FLR is an important limiting factor for major liver resections^20^,^21^. Thanks to the development of techniques including PVE or PVL^22^, the pool of candidates eligible to receive liver resection to treat primary or secondary liver tumors has been expanded. Unfortunately, sufficient FLR is not achievable in some patients, and there is a concern about stimulating tumor progression by PVE or PVL before liver resection^23^. A new two-step approach has recently been developed using the acronym ALPPS to describe this operation^24^. To the best of our knowledge, there are only several basic animal research studies on ALPPS^11^,^12^,^13^ to study the mechanisms of liver regeneration associated with this surgical procedure. In this study, a rat model of ALPPS has been successfully established, and the effects of LLL resection, transection, PVL and ALPPS on liver regeneration were studied. The results indicated that postoperative liver regeneration in the ALPPS group was significantly higher than that in all the other groups. Also, in the ALPPS group the expressions of Ki-67 and PCNA, the two important cell proliferation markers were much stronger than that in the other groups after surgery. The significant increase in the number of Ki-67- and PCNA-positive hepatocytes in the ALPPS group indicated a higher rate of hepatic regeneration in the ALPPS group. We have also investigated the effects of ALPPS on cell cycle and found that rapid induction of Cyclin D1 and Cyclin E, as well as their catalytic partners, Cdk2 and Cdk4, occurred after ALPPS in rats. Complexes containing cyclin E and Cdk2 assembled in the regenerating livers leading to increased Cdk2-associated kinase activity.

Although there are lots of ongoing debates regarding the mechanisms of the accelerated liver regeneration after ALPPS, the mechanism for this phenomenon is not clear and warrants detail mechanistic investigation. As reported, liver regeneration is an event that involves multiple cellular processes and has a complex interaction with cytokines and growth factors^14^,^15^. Schlegel *et al.* suggested that transection of the parenchyma may induce an inflammatory response with the release of putative growth factors, which may enhance the regenerative process^11^. This speculation was consistent with the results in our study, which indicated that both the mRNA and protein levels of TNF-α, lL-6 and HGF in the regenerating liver tissues were higher in the ALPPS group than that in the other groups. We speculated that the synergistic effects of PVL and transection might contribute to the improvements in the cytokine and growth factor responses. One explanation for the different responses between PVL and ALPPS groups might be related to a different hepatic microcirculation perfusion and hemodynamic changes of the portal flow. The transection might leads to the discontinuation of “cross portal” circulation between the normally perfused and deportalized liver parts, which decreased microcirculation perfusion of the right median lobe and, thereby causing injury or necrosis of liver cells. Secondly, the transecion itself can also serve as a kind of liver injury, which can promote the cytokine response. Meanwhile, the H&E staining showed that the necrosis areas of the right median lobes were larger in the ALPPS group compared with the PVL group and that the serum ALT and AST levels, which represented the hepatocyte damage, were also high in the ALPPS group. This might also suggested that the liver regeneration associated cytokine and growth factor responses after ALPPS is triggered by both the portal vein ligation and liver transection. Recently, some researchers have demonstrated that Hippo-YAP signaling is a significant factor in controlling organ size in mammals^17^. Several studies have also indicated that the transcriptional co-activator YAP is activated during liver regeneration, resulting in its nuclear localization and activation of a proliferative transcriptional program^18^,^19^. Consistent with the results of these previous studies, our results indicated that YAP protein levels were significantly increased, especially during the first three days post-surgery, and the increase was most obvious in the ALPPS group. Our finding of the persistently increased YAP levels suggested that YAP continues to play a role in the reorganization of liver architecture after the perioperative period of ALPPS.

In summary, we speculated that there might be several possible explanations for the highest liver regeneration response in the ALPPS group: a) after complete separation of the LML by PVL and *in situ* transection, the entire portal flow is redistributed towards the FLR, thus constituting a stimulus to regeneration; b) the local trauma caused by liver partition (transection) can also be a stimulus; c) a synergistic activation of the liver regeneration-associated signaling pathways, such as cytokines and transcription factors, contribute to the significant liver regeneration after ALPPS. At present, the mechanisms for rapid promotion of hepatic regeneration after ALPPS is not clear, and the procedure is still in its initial stage^25^,^26^,^27^. There is no universally accepted clinical application of this operation. The short-and long-term effects on biological behavior of tumor growth is still unclear. Although there is a clinical report suggesting that ALPPS is also feasible in liver fibrosis^28^, there is no solid data to support its use in liver fibrosis. The procedure still needs further clinical data, and more basic researches are urgently needed.

## Materials and Methods

### Animals

In this study, 480 male Sprague-Dawley rats weighing between 200 and 250 g (Laboratory Animal Center of the Chinese Academy of Sciences, Shanghai)were used. All the rats were provided with unlimited access to food and water before and after treatment. All surgeries were performed under anesthesia. The experimental protocol was reviewed and approved by the Committee on the Use of Live Animals in Teaching and Research of the Harbin Medical University, Harbin, China (SYSK 2010-012). We confirmed that all experiments were performed in accordance with relevant approved guidelines and regulations.

### Experimental design

In the current study, the rats were randomly divided into the sham group, the LLL resection group, the transection group, the PVL group, and the ALPPS group. The rats were fasted 12 h before the operations. All operations were performed under an operating microscope (Binocular Operation Microscope; Type GX.SS.22-3; Shanghai Medical Optical Instruments Co, Ltd. China). After anesthesia, the abdomen was opened through a transverse incision. The liver was dissected from its ligaments. Briefly for the ALPPS procedure, dissection of the caudate lobe followed by ligation of the portal vein supplying the caudate lobe with 7-0 silk were performed. The same procedure was then repeated on branches of the portal veins supplying the right and left lobes. Subsequent dissection and ligation of the right branch of the portal vein which supplied the median lobe was followed by a line of demarcation between the perfused and the unperfused parts of the median lobe. To complete Step I of the ALPPS procedure, transection of liver parenchyma along the demarcation line was carried out using a disposable drip bipolar coagulation forceps down to the anterior aspect of the inferior vena cava. In the PVL group, the rats were operated in the same way as the rats in the ALPPS group, with the exception of not carrying out transection of hepatic parenchyma along the demarcation line. The procedure of PVL was performed as shown in Fig. 1E. In the LLL resection group, only the left lateral lobe was resected. In the transection group, only transection of hepatic parenchyma was carried out along a transient ischemia demarcation line using a vascular clamp instead of ligation as in the ALPPS group. In the sham group, the hepatic arteries, portal veins and bile ducts were dissected but not ligated. The abdomens of the rats were closed using a double running suture. Intermittent Pringle’s maneuver was used on demand to control intraoperative blood loss.

The rats in all the groups underwent a relaparotomy 72 hours after Step 1. The caudate lobe, right lobe and right median lobe were resected, keeping only the left median lobe. The rats in each group were sacrificed on day 1, 2, 3, 5 and 7 after the second operation (n = 4 to 6 at each time point). Blood samples were collected from the inferior vena cava at each time point. They were centrifuged at 3000 g for 10 min, and the serum was stored at −80 °C until the tests for liver biochemical determinations were performed. After weighing, approximately 200 mg of liver tissues from the right and left median lobes were immediately frozen in liquid nitrogen and then stored at −80 °C. The remaining liver lobes were fixed in formaldehyde.

### Real-time polymerase chain reaction (PCR)

Total RNA was extracted from 50 mg of liver tissues using TRIzol reagent (Invitrogen, USA). Five micrograms of RNA were reverse-transcribed using the Thermoscript RT-PCR kit (Invitrogen), yielding the complementary DNA template. The quantitative RT-PCR amplification and the data analysis were performed using an ABI-Prism 7000 Sequence Detector system. The primers for IL-6, HGF, and TNF-α areshown in Table S1. The mRNA expression levels for each sample were normalized. The results obtained represented fold induction versus baseline levels in the control rats.

### ELISA for inflammatory Cytokine and HGF Response

Regenerating liver samples were homogenized in buffer (phosphate-buffered saline solution, pH 7.4) and centrifuged (10000 g; 4 °C; 10 min), and the supernatant was used for analysis. The concentrations of proteins in the liver tissues were measured with ELISA kits, IL-6 (R&D Systems, SR6000B), TGF-α (Enzyme-linked biotechnology company Shanghai Science and Technology Co.,Ltd., ml002895), NF-KB (Nanjing SenBeijia Biotechnology Co.,Ltd., SBJ-R0177), STAT3 (Abcam, ab126459), TNF-α(Abcam, ab46070), OSCF (Enzyme-linked biotechnology company Shanghai Science and Technology Co.,Ltd., ml002853), HGF (R&D Systems, MHG00), JAK (Enzyme-linked biotechnology company Shanghai Science and Technology Co., Ltd., ml028415), SOCS-3 (Shanghai KeMin Biotechnology Co., Ltd., E-EL-R0390), ERK1/2 (abcam, ab119674), c-met (Wuhan Huamei Biological Engineering Co., Ltd., CSB-E13491r), Src (Shanghai Beinuo Biotechnology Co., Ltd., USCN10438), Akt (R&D Systems, DYC1775E), c-myc (Beijing ShengShiZhongFang Biotechnology Co., Ltd., E-EL-R0995c), c-jun (R&D Systems, DYC1205-2) and YAP (Shanghai Gaochuang Biotechnology Co., Ltd., CSB-EL026244RA) according to the manufacturer’s instructions.

### AST and ALT Levels

The blood samples obtained from the inferior vena cava at different time points were immediately centrifuged at 2000 g for 5 minutes. Serum aspartate aminotransferase (AST) and alanineaminotransferase (ALT) levels were measured with a commercial assay kit (Nanjing Jiancheng Biological Technology, Inc., China) using a serum Hitachi 7020 automatic biochemical analyzer. Enzyme activities were expressed as an international unit per liter (U/L).

### Histological Examination

The liver tissues were immersion fixed in 4% formaldehyde, embedded, sectioned, and stained with hematoxylin-eosin (H&E) for the necrotic area determination. The liver sections were immunostained for Ki-67 (Santa Cruz Biotechnology, Inc.) or proliferating cell nuclear antigen (PCNA) (Santa Cruz Biotechnology, Inc.) as described previously^29^. The number of Ki-67-positive and PCNA-positive hepatocytes were determined in 10 random visual fields (20×). To determine the areas of necrosis, two calculating methods were used in the study: 1) The necrotic areas after PVL or ALPPS were quantified in 10 visual fields (100×) using the Carl Zeiss AxioVision 4 LE program (Carl Zeiss, Jena, Germany). All histological analyses were performed in a blinded fashion with respect to the experimental groups. 2) The necrotic areas of right median lobes were quantified in 10 random visual fields using Adobe Photoshop CS 5. Necrosis was expressed as the percentage of necrotic tissue: 0, no necrosis; 1, less than 25%; 2, 25%–50%; 3, 50%–75%; and 4, at least 75% necrosis.

### Western blotting

Standard western blot assays were used to analyze protein expression, as described previously^30^.The following antibodies against Yap, cyclin D1, cyclin E, STAT3, p-STAT3 (Tyr 705), and β-actin were obtained from the Cell signaling Biotechnology. The antibodies against Cdk2 and Cdk4 were obtained from the Abcam.

### Magnetic Resonance Imaging and Analysis

Images were acquired and analyzed as described previously^31^. Briefly, data acquisition was synchronized with the respiratory cycle of free breathing rats. Liver volume was determined by manual segmentation on each slice and summing as described^3^.

### Electrophoretic mobility shift assay (EMSA)

The detailed methodology has been described previously^2^. Briefly, nuclear extract (10 μg) was incubated with 1 μg of poly (deoxyinosinic-deoxycytidylic acid) in binding buffer for 30 min at 4 °C. DNA binding activity was confirmed with a biotin-labeled oligonucleotide bio-NF-κB probe.

### Immunoprecipitation and histone H1 kinase assay (Cdk2 activity) assays

Immunoprecipitation and histone H1 kinase assay (Cdk2 activity) were performed as previously described^32^.

### Statistics

All data are expressed as means ± standard deviation (SD). Differences between the groups were assessed by one-way or two-way analysis of variance (ANOVA, Bonferroni post-test), using an appropriate post-hoc comparison test. A significant difference was assumed when *P* was smaller than 0.05. Statistics were performed using the software package GraphPad 4.0 (GraphPad Software Inc., San Diego, CA) and SPSS 12.0.1 (SPSS Inc., Chicago, IL).

## Additional Information

**How to cite this article**: Shi, H. *et al.* A preliminary study of ALPPS procedure in a rat model. *Sci. Rep.* **5**, 17567; doi: 10.1038/srep17567 (2015).

## Supplementary Material

Supplementary Information
