# Supplementary material for: A preliminary study of ALPPS procedure in a rat model
Source: Sci Rep. 2015 Dec 3;5:17567. doi: 10.1038/srep17567 (PMC4668375; doi:10.1038/srep17567)

SREP-15-17599B

A preliminary study of the ALPPS procedure in a rat model

Huawen Shi*[1], Guangchao Yang*[1], Tongsen Zheng[†1], Jiabei Wang[1], Lulu Li[2], Yingjian Liang[1], Changming Xie[1], Dalong Yin[1], Boshi Sun[1], Jing Sun[1], Huanlai Wang[1,3], Shangha Pan[1], Hongchi Jiang[1], WanYee Lau[†4], Lianxin Liu[†1]

Disclosures: The paper is not based on a previous communication to a society or meeting (with full details) and the authors declare no conflicts of interest.

Supplemental Data

Supplementary Tables

Table SI Changes in protein levels of genes related to liver regeneration in rats.

Changes in protein levels of genes related to liver regeneration in rats.

	Day 2 after Step I					Day 2 after Step II				
Gene	Sham	Transection	LLL Resection	PVL	ALPPS	Sham	Transection	LLL Resection	PVL	ALPPS
IL-6	1.00±0.21	3.45±0.45	2.45±0.67	4.62±0.34	18.2±0.15'	1.00±0.11	2.41±0.18	2.48±0.78	3.89±0.41	15.4±0.57*
TGF-α	1.00±0.23	1.56±0.34	1.72±0.41	2.13±0.23	3.28±0.45	1.00±0.15	2.13±0.34	2.67±0.23	3.12±0.22	4.11±0.34
NF-κB	1.00±0.15	4.34±0.56	2.67±0.43	5.31±0.45	9.42±0.23*	1.00±0.21	3.45±0.41	2.16±0.32	4.67±0.37	14.4±0.37*
STAT3	1.00±0.09	3.12±0.67	2.19±0.32	3.78±0.36	12.4±0.17*	1.00±0.05	3.41±0.23	2.56±0.12	3.56±0.31	9.45±0.77*
TNF-α	1.00±0.27	3.78±0.23	4.12±0.67	3.78±0.28	8.45±0.28*	1.00±0.17	2.67±0.43	3.56±0.43	3.15±0.21	6.34±0.31
SCF	1.00±0.13	2.12±0.15	1.67±0.35	1.79±0.48	2.56±0.25	1.00±0.14	1.76±0.22	1.78±0.34	1.67±0.34	3.78±0.21
EGF	1.00±0.16	5.12±0.34	2.13±0.45	3.14±0.31	10.4±0.21*	1.00±0.32	3.78±0.34	3.12±0.23	4.14±0.23	12.3±0.66*
HGF	1.00±0.06	2.34±0.35	3.17±0.28	4.23±0.42	11.2±0.32*	1.00±0.25	3.12±0.21	4.13±0.25	4.19±0.45	13.1±0.31*
JAK	1.00±0.14	1.45±0.34	1.78±0.31	2.11±0.23	3.23±0.18	1.00±0.08	1.72±0.35	1.67±0.26	2.34±0.26	3.45±0.34
SOCS-3	1.00±0.17	3.13±0.16	2.12±0.56	3.13±0.26	4.13±0.27	1.00±0.07	2.77±0.27	1.87±0.45	3.56±0.33	5.12±0.11
ERK-1/2	1.00±0.12	2.67±0.45	3.12±0.31	4.23±0.19	9.43±0.81*	1.00±0.14	3.28±0.33	3.56±0.12	4.67±0.41	8.54±0.36*
C-kit	1.00±0.04	1.87±0.32	1.89±0.51	2.21±0.31	3.67±0.56	1.00±0.11	2.13±0.28	2.01±0.32	2.34±0.38	4.15±0.46
c-met	1.00±0.05	1.34±0.19	1.78±0.67	1.84±0.28	2.42±0.33	1.00±0.06	1.77±0.34	1.76±0.15	1.93±0.21	2.56±0.18
Src	1.00±0.16	2.56±0.43	2.04±0.32	2.91±0.42	3.14±0.28	1.00±0.09	3.14±0.22	2.45±0.25	3.11±0.34	4.23±0.34
Akt	1.00±0.18	1.69±0.28	1.72±0.41	2.13±0.38	2.89±0.46	1.00±0.08	1.56±0.32	1.89±0.37	2.44±0.44	2.11±0.15
c-myc	1.00±0.04	2.14±0.67	2.29±0.67	1.78±0.89	2.56±0.51	1.00±0.12	1.55±0.38	2.13±0.34	1.89±0.23	2.54±0.34
c-jun	1.00±0.16	1.34±0.41	1.67±0.51	2.45±0.31	3.13±0.34	1.00±0.16	2.11±0.33	1.87±0.26	2.56±0.25	3.56±0.21
YAP	1.00±0.31	2.87±0.34	3.12±0.46	4.67±0.23	23.4±0.78*	1.00±0.11	2.24±0.17	3.45±0.33	5.21±0.32	21.3±0.62*

Notes: Values represent changes of protein expression in different groups. *$p<0.001$vs PVL group.

Table S2 Primer sequences for Real-time PCR assays of IL-6, TNF-α, HGF and GAPDH.

Gene	Forward	Reverse
IL-6	ACAGCGATGATGCACTGTCAG	ATGGTCTTGGTCCTTAGCCAC
TNF-α	AAATGGGCTCCCTCTCATCAGTTC	TCTGCTTGGTGGTTTGCTACGAC
HGF	TCCTGTGCCAAAACAAAACA	GGTGCTGACTGCATTTCTCA
GAPDH	ACCACAGTCCATGCCATCAC	TCCACCACCCTGTTGCTGTA

Shi et al; Supplementary Figure Legends

Figure S1 Changes in the right side of the middle liver lobe weight/body weight ratios after operations in the different groups. Mean ± SD. (*n* = 4–6 animals per group at each time point).

Figure S2 Changes of the body weight of the animals during the period. Mean ± SD. (*n* = 4–6 animals per group at each time point).

Figure S3 Cdk2-associated kinase activity after ALPPS. (A) Liver protein extracts (400 pg/lane) from Sham or ALPPS rats were immunoprecipitated with anti-Cdk2 antibody, and the immune complexes were assayed for kinase activity with histone H1 as a substrate. **(B)** Densitometric analysis of phosphorylated histone H1 in A after subtracting nonspecific activity (expressed as percent of time-matched Sham controls).

Figure S4 Comparison of representative liver regeneration-associated gene expressions in the regenerating liver tissues. Gene expression detected by Real-time PCR at day 0, day 1, day 2 and day 5 was normalized by GAPDH. Data are means ± SD of six animals in each group. *** P＜0.001, ** P＜0.01 compared with the PVL, transection and LLL resection groups.

Shi et al; Supplementary figures

Figure S1

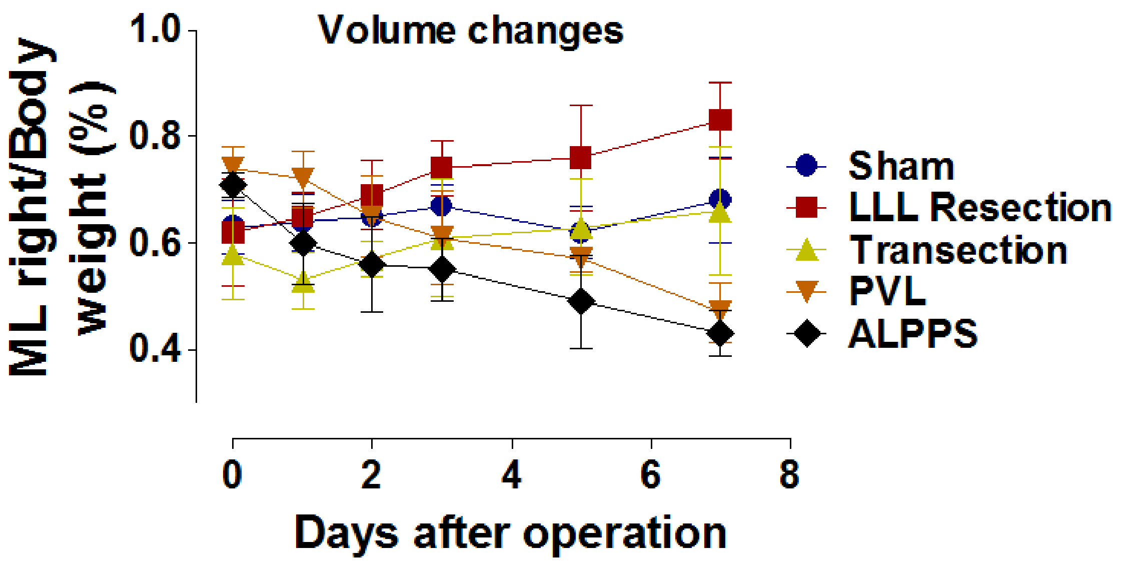

Figure S2

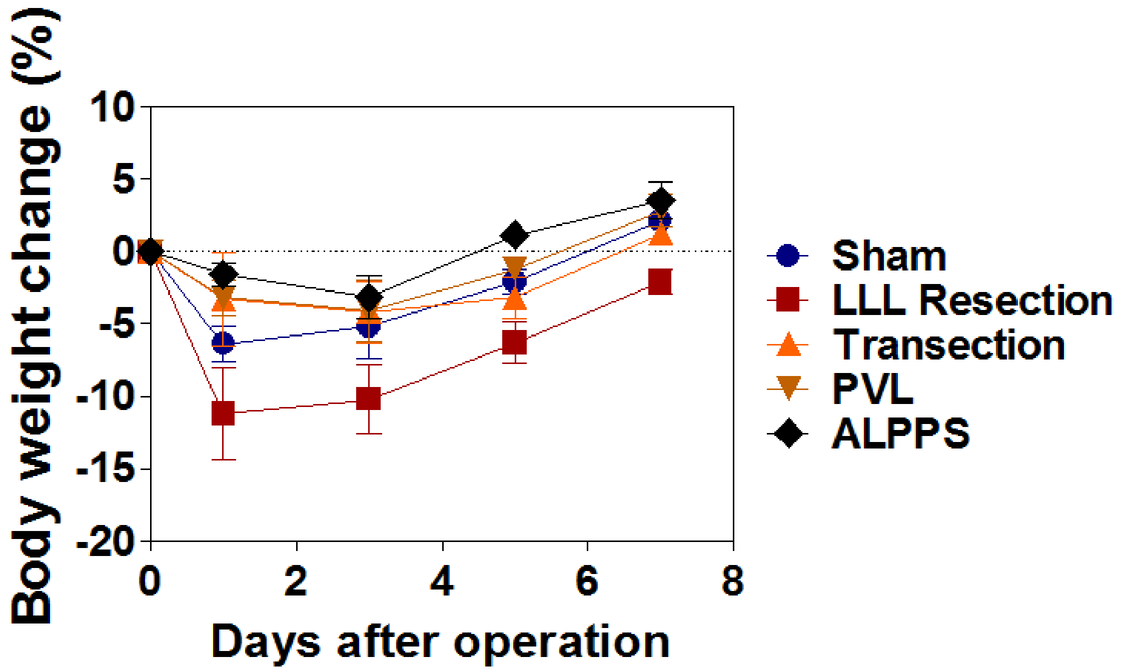

Figure S3

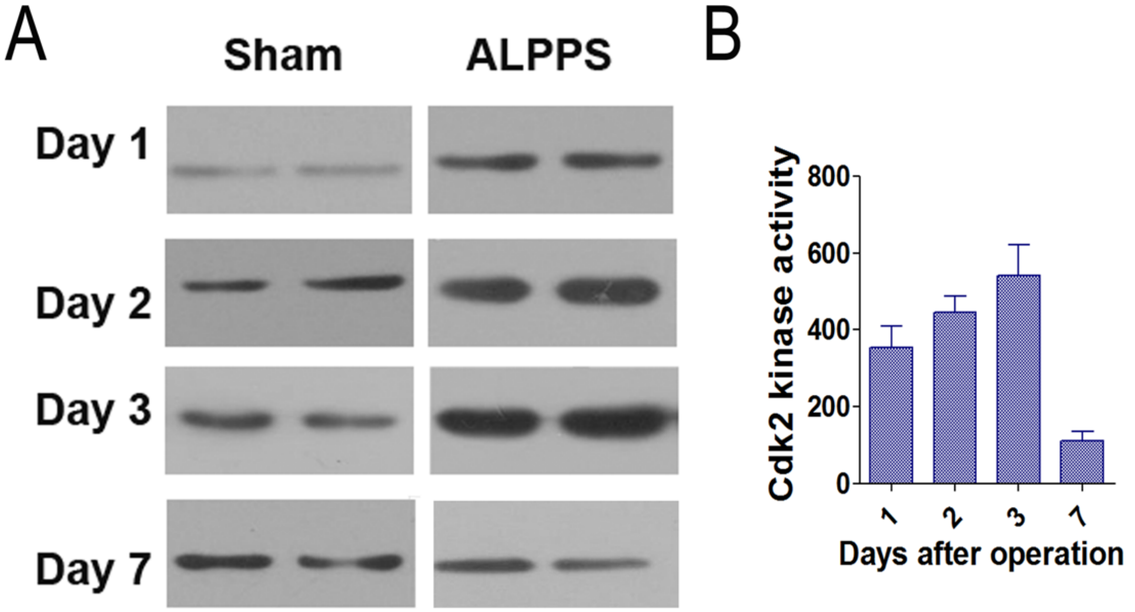

Figure S4

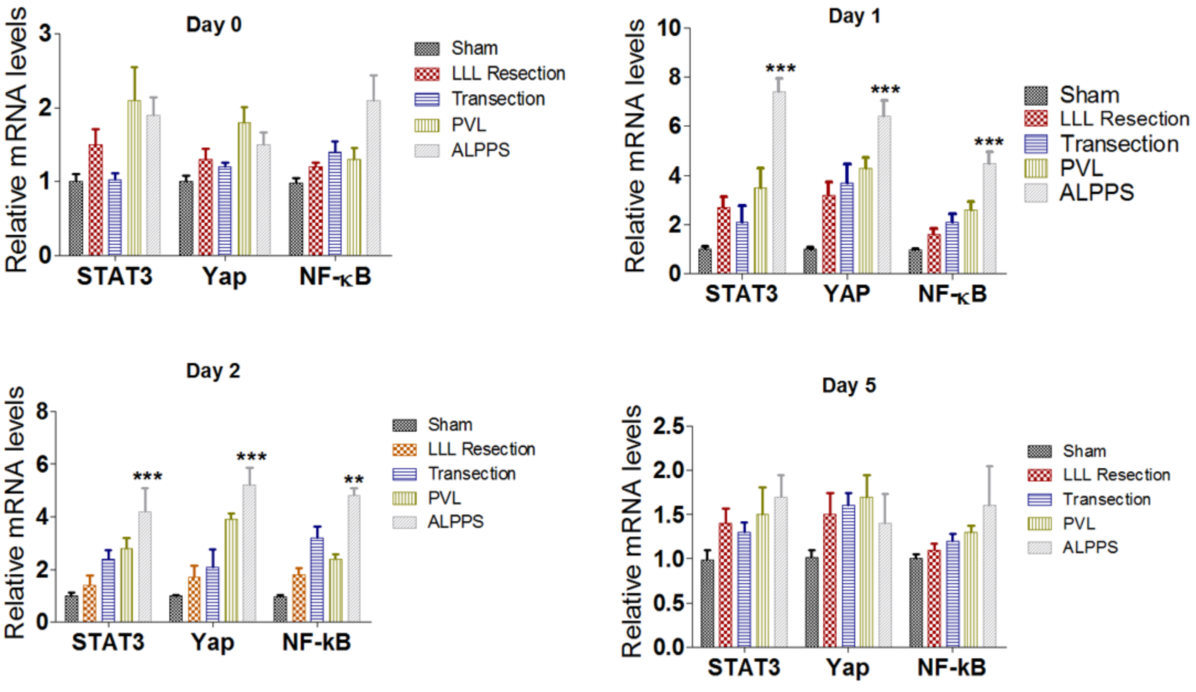

Supplement: Supplementary Information [file srep17567-s1.pdf]
